# Genetic Determinants of Cellular Homeostasis Contributing to the Risk of Type 2 Diabetes

**DOI:** 10.32607/actanaturae.27855

**Published:** 2026

**Authors:** Y. R. Timasheva, O. V. Kochetova, T. R. Nasibullin, T. M. Kochetova, Z. Balkhiyarova, G. F. Korytina, A. Nouwen

**Affiliations:** Institute of Biochemistry and Genetics, Ufa Federal Research Centre, Russian Academy of Sciences, Ufa, 450054 Russia; Bashkir State Medical University, Ufa, 450008 Russia; Section of Statistical Multi-Omics, Department of Clinical & Experimental Medicine, School of Biosciences & Medicine, University of Surrey, Guildford, GU2 7XH, UK; Department of Psychology, Middlesex University, London, NW4 4BT, UK

**Keywords:** Type 2 diabetes, polygenic risk score, oxidative stress, insulin signaling

## Abstract

Genetic variation in the genes involved in the maintenance of cellular homeostasis may influence susceptibility to type 2 diabetes (T2D). However, the nature of the interplay among these loci and their prognostic value remains unclear. In this study, we analyzed variants in the FOXO3A, FOXO3, FOXO1, HMOX1, and SIRT1 genes. The HMOX1 rs2071746*T/T genotype was associated with an increased risk of T2D (OR = 1.36, PFDR = 0.030), whereas the FOXO1 rs9549240 (OR = 0.52, PFDR = 0.002) and SIRT1 rs3758391 (OR = 0.80, PFDR = 0.015) genotypes exhibited a protective effect. We detected nonlinear interactions, including FOXO1 rs9549240*G + SIRT1 rs3758391*T + SIRT1 rs7895833*A and HMOX1 rs2071746*A + SIRT1 rs3758391*T + SIRT1 rs7895833*A, with significant synergistic effects (SF = 3.19 and 2.56, P < 0.03). Models incorporating these interactions achieved an AUC of 69.8%, which increased to 86.2% when combined with age, sex, and the body mass index. These findings suggest that interactions between the pathways regulating oxidative stress and metabolism may contribute to the genetic predisposition to T2D.

## INTRODUCTION

Type 2 diabetes (T2D) is a complex, non-autoimmune disorder characterized by insulin resistance and progressive β-cell dysfunction, with impaired insulin secretion playing a key role in the development of hyperglycemia [1]. While the mechanisms of β-cell dysfunction are not fully understood, genetic factors are known to modulate the balance between insulin secretion and insulin resistance. T2D is predominantly polygenic in nature, with common alleles exerting modest cumulative effects and rare mutations accounting for only a small number of cases.

The FOXO (Forkhead box class O) protein family regulates metabolism and homeostasis in insulinsensitive tissues, including liver, β-cells, and adipose tissue [2]. Through the PI3K/Akt signaling pathway, FOXO proteins influence carbohydrate and lipid metabolism, fasting adaptation, and cell survival [3]. FOXO1 also controls appetite: its hypothalamic overexpression promotes hyperphagia under cerebral insulin resistance [4]. In β-cells, FOXO1 improves insulin sensitivity and reduces hepatic gluconeogenesis [5]. Although animal studies strongly support its involvement in the metabolic effects of insulin, human data remain ambiguous: some FOXO1 haplotypes are associated with a reduced risk of developing T2D, while others demonstrate no significant effect [2].

Sirtuins, a family of NAD^+^-dependent deacetylases, regulate DNA repair, metabolism, inflammation, and aging. SIRT1, expressed predominantly in metabolically active tissues, controls glucose and lipid metabolism, mitochondrial biogenesis, and insulin sensitivity [6, 7]. SIRT1 gene variants are linked with obesity and T2D, with certain single nucleotide polymorphisms (SNPs) being associated with reduced insulin secretion in high-risk individuals [8]. Reduced SIRT1 expression is also observed in ischemic heart disease, and specific variants of this gene have been shown to influence individual cardiovascular risk [9].

Heme oxygenase-1 (HMOX-1) plays a key role in maintaining cellular homeostasis by catalyzing heme degradation into biliverdin, iron, and carbon monoxide, conferring pronounced cytoprotective and antiinflammatory effects [10]. Stress-induced upregulation of HMOX1 limits oxidative damage, and the HMOX1 rs2071746 polymorphism has been previously associated with a wide range of pathological conditions [11].

In this study, we evaluated genetic variants in the FOXO, SIRT1, and HMOX1 genes, assessing their associations with T2D, as well as the gene–gene interactions and polygenic effects contributing to the development of the disease.

## EXPERIMENTAL

**Study Sample**

This study was conducted in compliance with the principles of the Declaration of Helsinki and approved by the Local Ethical Committee of the Institute of Biochemistry and Genetics, Ufa Federal Research Centre of the Russian Academy of Sciences, Ufa, Russian Federation (Protocol No. 8, March 14, 2012). Written informed consent was obtained from all the participants.

DNA samples were collected from unrelated individuals of Tatar ethnicity residing in the Republic of Bashkortostan between 2012 and 2024. The study group included 643 patients with T2D (164 men and 479 women), and 448 control individuals (249 men and 199 women). The study design, inclusion and exclusion criteria, baseline characteristics of the participants, as well as methods for anthropometric and biochemical measurements have been described previously [12, 13].

**Genotyping**

DNA was extracted from peripheral blood leukocytes using the standard phenol–chloroform procedure. Genotyping was performed by allelic discrimination using real-time PCR (Bio-Rad CFX96 system, Bio-Rad Laboratories Inc., USA) with TaqMan Assays (Thermo Fisher Scientific). Quality control included re-genotyping 5% of samples, with complete concordance. The criteria for including loci in the study were based on the functional significance of polymorphisms, as assessed using the RegulomeDB v. 2.2 database [14] and HaploReg v3 resource [15]. Additionally, we considered data on associations or linkage with variants related to diabetes and metabolic traits from genome-wide (GWAS) and/or candidate gene association studies. Furthermore, a minor allele frequency (MAF) threshold of no less than 0.05 was applied.

**Statistical Analyses**

Genotype and allele frequencies were tested for Hardy–Weinberg equilibrium. Associations between polymorphisms and T2D risk were assessed under five inheritance models (dominant, recessive, codominant, log-additive, and overdominant) using the SNPassoc R package (v2.1-0) [16]. The inclusion of the overdominant model allowed for consideration of potential heterozygote advantage.

Multi-locus associations were analyzed with APSampler software (v3.6.0) (http://apsampler.sourceforge.net/) [17], which is based on the Markov Chain Monte Carlo (MCMC) stochastic approach to identifying enriched allele combinations. All seven variants, even those in linkage disequilibrium (LD), were included in the analysis, to capture possible gene–gene interactions. Additional tests confirmed that the exclusion of one correlated SIRT1 SNP did not affect the association with FOXO1 rs9549240. Allelic combinations were visualized using Euler–Venn diagrams (eulerr R package), where overlaps represented interactions and color gradients reflected odds ratio ratios (ORR). Multiple testing correction was performed using the Benjamini–Hochberg false discovery rate (FDR) method [18].

Nonlinear interactions were further evaluated using synergy factor (SF) analysis [19]. Predictive models were built with stepwise multivariate logistic regression (IBM SPSS v22.0), including individual variants and significant allele pairs identified by SF analysis.

Polygenic risk scores (PRSs) were calculated from the best-fitting model for each variant, weighting risk alleles by ORs from age- and sex-adjusted logistic regression. When OR < 1.0, the alternative allele was used as reference. Variants in LD on the same chromosome were excluded. Principal component analysis (prcomp in R) was used to correct for population structure.

The predictive value of PRS was evaluated by ROC analysis, with performance quantified as the area under the curve (AUC). Models were implemented using Epi: Statistical Analysis in Epidemiology [20] and pROC [21] R packages. Predictive accuracy was tested by 10-fold cross-validation (caret package) and by non-parametric bootstrapping (1,000 resamples), yielding mean AUC and 95% confidence intervals.

## RESULTS

**Table 1:** The results of the association analysis of the studied loci with type 2 diabetes

Chr: Position	Gene	SNP	MA	MAF		P_HWE_	Model	OR(95%CI_OR_)	P	P_FDR_
				Control	T2D					
6:108567390	FOXO3A	rs2253310	G	0.27	0.24	0.342	Overdominant C/C-G/G vs. C/G	0.67 (0.52-0.86)	0.002	0.003
6:108677063	FOXO3	rs3800231	A	0.43	0.39	0.066	Overdominant G/G-A/A vs. A/G	0.63 (0.50-0.81)	2.63×10^-4^	9.23×10^-4^
10:67863299	SIRT1	rs7895833	G	0.31	0.28	0.058	Recessive A/A-G/A vs. G/G	0.78 (0.52-1.17)	0.233	0.233
10:67883584	SIRT1	rs3758391	C	0.49	0.43	0.107	log-Additive 1,2,3	0.80 (0.68-0.95)	0.011	0.015
10:67907144	SIRT1	rs3818292	G	0.23	0.18	0.18	Overdominant A/A-G/G vs. A/G	0.62 (0.47-0.80)	2.64×10^-4^	9.23×10^-4^
13:40612507	FOXO1	rs9549240	T	0.37	0.31	0.127	Recessive G/G-T/G vs. T/T	0.52 (0.35-0.76)	6.77×10^-4^	0.002
22:35380679	HMOX1	rs2071746	T	0.43	0.47	0.563	Dominant T/T vs. A/T-A/A	1.36 (1.04-1.78)	0.026	0.030

Note: Chr - chromosome

Position - genomic position according to Genome Reference Consortium Human Build 38 (GRCh38);

SNP - single nucleotide polymorphism;

MA - minor allele;

MAF - minor allele frequency;

P_HWE_ - Hardy-Weinberg equilibrium p-value;

OR - odds ratio;

95%CI_OR_ - 95% confidence interval for the odds ratio;

P - level of significance;

P_FDR_ - p-value with the Benjamini-Hochberg adjustment.

We analyzed associations between T2D and seven loci within the FOXO3A, FOXO3, FOXO1, HMOX1, and SIRT1 genes, identifying significant associations for five of them (Table 1). The HMOX1 rs2071746*T/T genotype was associated with an increased T2D risk (dominant model, OR =  1.36, PFDR = 0.030), while the FOXO1 rs9549240 (recessive model, OR =  0.52, PFDR = 0.002) and SIRT1 rs3758391 (log-additive model, OR = 0.80, PFDR = 0.015) variants exhibited a protective effect. SIRT1 rs3818292, FOXO3 rs3800231, and FOXO3A rs2253310 also conferred reduced T2D risk (overdominant model).

These variants were used to construct PRS, excluding correlated SNPs (FOXO3A rs2253310 with FOXO3 rs3800231, r² = 0.628; and SIRT1 rs3758391 with rs3818292 and rs7895833, r² = 0.145 and 0.524). The final PRS model included rs2253310, rs3758391, rs9549240, and rs2071746, weighted by OR.

Internal validation via 10-fold cross-validation yielded a mean AUC of 57.5% (sensitivity 17%, specificity 90%). Bootstrapping (1,000 resamples) confirmed the stability of the model (AUC 57.5%, 95% CI: 54–61%). PRS values were higher in T2D cases than in controls (6.22 ± 0.07 vs. 5.75 ± 0.08, P = 1.11 × 10^-5^) and were associated with an increased T2D risk (OR = 1.17 [1.09–1.26], P = 1.04 × 10^-5^).

**Fig. 1:**
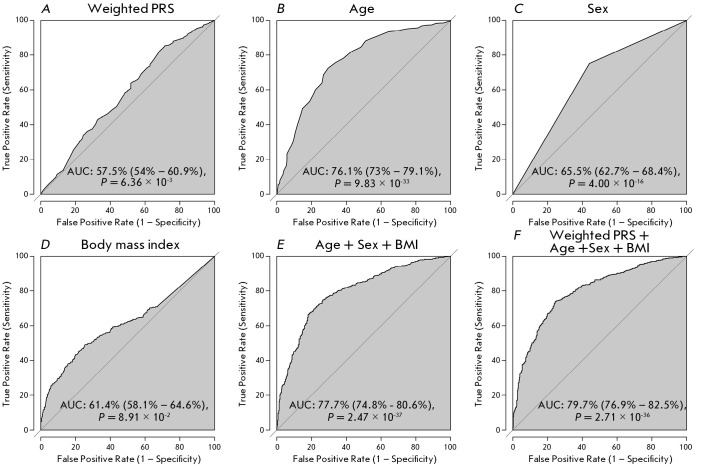
ROC curves illustrating the performance of various models for predicting type 2 diabetes. The models include: (A) weighted polygenic risk scores; (B) age; (C) sex; (D) body mass index (BMI); (E) sex + age + BMI; and (F) weighted polygenic risk scores + sex + age + BMI. AUC (Area Under the ROC Curve) indicates the discriminatory power of the model: 90–100% – excellent; 80–90% – very good; 70–80% – good; 60–70% – fair; and 50–60% – poor

ROC analysis showed modest predictive power for PRS alone (AUC = 57.2–57.5%); however, the inclusion of BMI (61.4%), sex (65.5%), and age (76.1%) significantly enhanced the predictive ability, increasing the AUC to 79.7% (Fig. 1).

A comparison of the logistic models revealed that the PRS alone served as a significant predictor of T2D (P = 0.006), although its discriminatory power remains limited. Clinical covariates taken together demonstrated high significance (P < 1 × 10- ³ 6 ), while the combined model (PRS + age + sex + BMI) provided the optimal predictive power (ΔDeviance = 6, ΔAIC = 4). This indicated the incremental prognostic value of the genetic component over standard clinical risk factors.

**Fig. 2:**
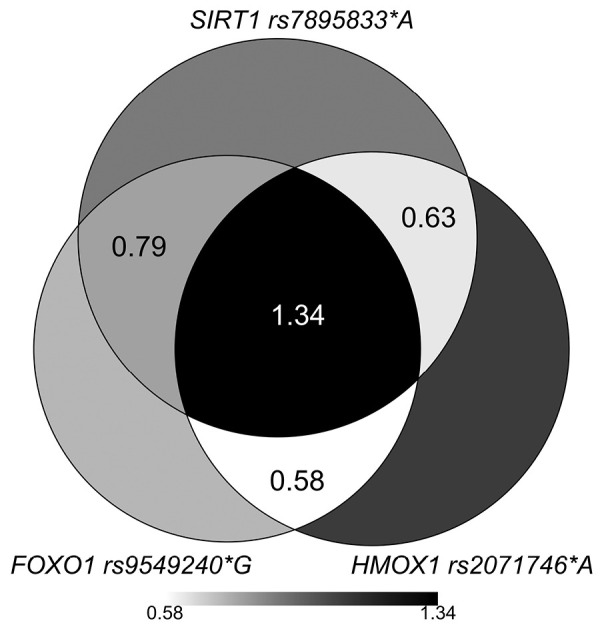
The Euler–Venn diagram illustrating the FOXO1 rs9549240*G, HMOX1 rs2071746*A, and SIRT1 rs7895833*A combination associated with type 2 diabetes. The circles represent individual variants, while the overlaps indicate interactions. The color gradient reflects the synergy between components, calculated as the ratio of the observed odds ratio (OR) to the product of the individual odds ratios (OR)

Using the APSampler tool, we identified key multilocus combinations associated with T2D, including FOXO1 rs9549240G + SIRT1 rs3758391T + SIRT1 rs7895833A (OR  =  1.93, PFDR =  5.94  ×  10-5), and HMOX1 rs2071746A + SIRT1 rs3758391T + SIRT1 rs7895833A (OR =  1.87, PFDR = 3.14 × 10-5) (Fig. 2, Table 2).

**Table 2:** The results of the analysis of the association of the combinations of the studied loci with type 2 diabetes

Pattern	Controls, %	T2D, %	OR	95%CIOR	P	PFDR
FOXO1 rs9549240*G + SIRT1 rs3758391*T + SIRT1 rs7895833*A	0.58	0.72	1.93	1.48–2.50	5.45×10^-7^	5.94×10^-5^
HMOX1 rs2071746*A + SIRT1 rs3758391*T + SIRT1 rs7895833*A	0.44	0.59	1.87	1.45–2.40	5.77×10^-7^	3.14×10^-5^
FOXO1 rs9549240*G + HMOX1 rs2071746*A + SIRT1 rs7895833*A	0.50	0.64	1.80	1.40–2.31	2.94×10^-6^	1.07×10^-4^
SIRT1 rs3758391*T + SIRT1 rs7895833*A	0.67	0.78	1.82	1.38-2.41	1.55×10^-5^	4.23×10^-4^
FOXO1 rs9549240*G + SIRT1 rs7895833*A	0.76	0.84	1.74	1.28-2.37	3.09×10^-4^	0.002
FOXO1 rs9549240*G + SIRT1 rs3818292*A + SIRT1 rs7895833*A	0.72	0.81	1.70	1.27–2.29	2.44×10^-4^	0.001
FOXO3 rs3800231*A + SIRT1 rs3818292*A + SIRT1 rs3758391*C	0.49	0.36	0.60	0.47–0.77	3.90×10^-5^	0.001
FOXO3A rs2253310*C + SIRT1 rs3818292*G	0.40	0.28	0.59	0.46-0.77	4.85×10^-5^	0.001
FOXO3A rs2253310*G + SIRT1 rs3758391*C	0.37	0.26	0.59	0.45-0.77	6.26×10^-5^	0.001
FOXO3 rs3800231*A + FOXO3A rs2253310*C + SIRT1 rs3818292*A	0.65	0.53	0.62	0.48–0.79	9.52×10^-5^	0.001
FOXO1 rs9549240*T + SIRT1 rs3818292*G	0.23	0.14	0.54	0.39-0.74	9.94×10^-5^	0.001
HMOX1 rs2071746*T + SIRT1 rs3818292*G	0.36	0.26	0.61	0.47-0.79	1.47×10^-4^	0.001
FOXO3 rs3800231*A + SIRT1 rs3758391*C	0.50	0.39	0.64	0.50-0.82	2.33×10^-4^	0.001

Note: OR - odds ratio;

95%CIOR - 95% confidence interval for the odds ratio;

P - level of significance;

PFDR - level of significance with the Benjamini-Hochberg adjustment.

**Fig. 3:**
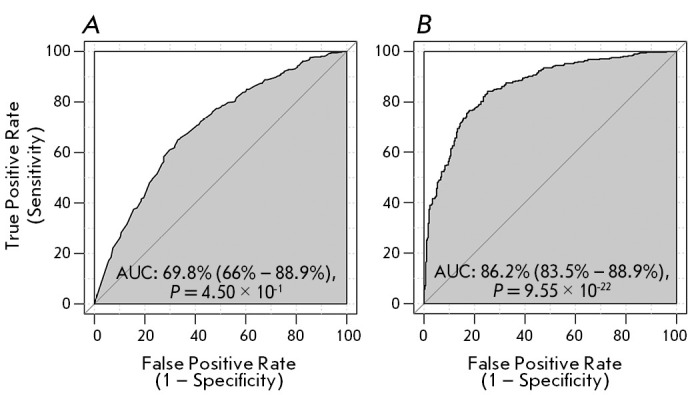
ROC curves demonstrating predictive performance for type 2 diabetes. (A) The model based on the studied genetic loci and their combinations. (B) The model incorporating genetic loci, their combinations, and clinical factors (age, sex, and body mass index)

Nonlinear interaction analysis revealed significant synergistic effects between SIRT1 rs3758391*T and SIRT1 rs7895833*A (SF = 3.19, P = 0.009), as well as FOXO1 rs9549240*G and HMOX1 rs2071746*A (SF = 2.56, P = 0.027). A multivariate logistic model including these variants and synergistic pairs achieved AUC = 69.8%, improving to AUC = 86.2% with added clinical variables (Fig. 3, Table 3).

**Table 3:** Logistic regression coefficients for a multifactorial model of genetic risk in type 2 diabetes

Predictor	Beta	SE	OR	95%CI_OR_	P
Sex	-0.50	0.19	1.66	1.14-2.41	0.008
Age	0.11	0.01	1.11	1.04-1.01	5.59 × 10^-23^
BMI	0.04	0.01	1.05	1.01-1.06	0.004
FOXO3 rs3800231	-0.65	0.18	0.52	0.37-0.74	0.001
HMOX1 rs2071746	-1.36	0.39	0.26	0.12-0.55	0.001
SIRT1 rs3818292	-0.51	0.19	0.60	0.41-0.88	0.008
SIRT1 rs3758391*T + SIRT1 rs7895833*A	0.76	0.19	2.14	1.46-3.13	0.001
FOXO1 rs9549240*G + HMOX1 rs2071746*A	1.38	0.37	3.97	1.93-8.18	0.001

Note: Beta - regression coefficient;

SE - standard error;

OR - odds ratio;

95%CIOR - 95% confidence interval for the odds ratio;

P - level of significance.

## DISCUSSION

In this study, we analyzed variants within the FOXO, SIRT1, and HMOX1 genes regarding their potential contribution to T2D susceptibility in a Tatar population. We identified associations with T2D for both individual variants and their nonlinear combinations. Using APSampler, we identified combinations significantly associated with the disease that exhibited substantial synergy between their components, including SIRT1 rs3758391*T + SIRT1 rs7895833*A (SF = 3.19, P = 0.009), and FOXO1 rs9549240*G + HMOX1 rs2071746*A (SF = 2.56, P = 0.027). These findings highlight a potential role for interactions between oxidative stress, transcriptional regulation, and metabolic pathways in the pathogenesis of T2D. However, most of the variants studied do not have confirmed associations in GWAS, which limits the generalizability of the findings. While the selected candidate gene approach allows for identification of biologically motivated signals, it requires external replication to confirm the robustness of the observed effects.

Assessment of the predictive value of the genetic data revealed moderate strength for the PRS calculated from individual loci associated with T2D. The improvement in predictive power upon the inclusion of the identified combinations demonstrates the potential for gene–gene interactions. However, the overall contribution of the genetic component remains limited compared to clinical characteristics. The most effective model was the one integrating both genetic and clinical data (AUC = 86.2%, 95% CI: 83.5–88.9%, P = 9.55 × 10- ²²), although the increase in accuracy was primarily driven by age, sex, and body mass index. In the absence of external validation, such models require cautious interpretation due to the risk of overfitting and the potential influence of cryptic population structure.

Several variants, including FOXO1 rs9549240, FOXO3 rs2253310/rs3800231, SIRT1 rs3818292/ rs3758391, and HMOX1 rs2071746, were also associated with BMI and obesity, the established risk factors for T2D. FOXO1 rs9549240 is in LD with a T2Dassociated haplotype (rs2701891–rs7337995) and may alter HIF-1 binding, potentially affecting hypoxia responses already disrupted in T2D [22]. Such findings are consistent with the biological functions of the FoxO family, which is involved in regulating energy metabolism, oxidative stress, inflammatory cascades, and DNA repair systems.

The associations with SIRT1 variants are particularly relevant, given the pivotal role of sirtuins in the carbohydrate and lipid metabolism [23]. SIRT1 rs3758391 conferred protection against T2D, while functional evidence suggests its potential effects on transcription factor binding [24, 25]. These variants are linked to regulation of inflammation, stress response, and β-cell survival [26, 27, 28, 29]. The observed interaction with the FOXO1 and HMOX1 loci further underscores the role played by SIRT1 in integrating the metabolic and stress-dependent pathways.

Associations with FOXO1 and FOXO3 variants support the importance of FoxO transcription factors in the pathogenesis of T2D. Associations between FOXO3 and BMI, as well as the risk of cardiovascular complications, have been described, suggesting a potential indirect influence on diabetes development through inflammatory mechanisms [30]. It is well established that FoxO is regulated by phosphorylation and acetylation [31, 32, 33]. Specifically, SIRT1 deacetylates FoxO, thereby enhancing antioxidant defense and DNA repair mechanisms. These biochemical signals may provide a basis for the combined genetic effects identified in our study. Despite conflicting data on the role of FoxO in the development of metabolic disorders across various studies [34, 35, 36, 37], our observations align with the hypothesis that FoxO modulates the inflammatory response, including the suppression of NF-κB activation and reduction of IL-6 and TNF-α production [38, 39, 40].

The identified association of HMOX1 rs2071746 with T2D underscores the involvement of oxidative stress in its pathogenesis. The HMOX1 rs2071746*A allele enhances promoter activity, whereas the T allele is associated with lower expression [41] and, as shown in our findings, an increased risk of T2D (OR = 1.36, PFDR = 0.030). Reduced HMOX1 expression may weaken cellular defense against oxidative and inflammatory damage [42, 43, 44], being consistent with earlier reports of impaired antioxidant defense systems in T2D [45, 46].

Results from the GTEx database analysis corroborate the functional significance of the observed associations: the FOXO1 rs9549240*G allele is characterized by higher expression in blood, adipose tissue, and brain, while SIRT1 rs3758391*T shows increased expression in metabolically active tissues (GTEx, September 19, 2025). The specific expression profiles within the central nervous system suggest a potential influence on appetite regulation and glucose homeostasis.

Overall, our findings indicate that variants in the FOXO, SIRT1, and HMOX1 genes may modulate the risk of T2D by influencing insulin signaling, oxidative stress, and inflammation. Nevertheless, the interpretation of these results is constrained by the lack of external replication and the use of a candidate gene approach. Follow-up studies in independent cohorts are needed to confirm the observed effects and assess their stability. Despite these limitations, our observations highlight the value of integrating molecular and clinical data to better understand the risk factors of T2D and develop more accurate predictive models.

## CONCLUSION

Our study identified associations between variants in the HMOX1, FOXO3, FOXO1, and SIRT1 genes and susceptibility to T2D in a Tatar population. The HMOX1 rs2071746*T/T genotype was found to be associated with an increased risk of T2D, whereas FOXO1 rs9549240 and SIRT1 rs3758391 exhibited protective effects. The identified combinations of variants at the FOXO1, SIRT1, and HMOX1 loci suggest the potential involvement of interactions between oxidative stress regulation and metabolic pathways in the formation of genetic predisposition to the disease. A multivariate model incorporating genetic data alongside age, sex, and BMI had a high predictive accuracy (AUC 86.2%). However, most of its predictive power is determined by clinical parameters. These findings highlight the potential role of the FOXO1, SIRT1, and HMOX1 genes in metabolic regulation and T2D pathogenesis, although they require confirmation in independent cohorts due to the limitations of the candidate gene approach. Given the contribution of gene–gene interactions and the influence of clinical factors, further integration of molecular and clinical data represents a promising avenue for more precise disease risk assessment.

## Abbreviations

AMPK/JNK: AMP-activated protein kinase/c-Jun N-terminal kinase
AUC: area under the curve
FDR: false discovery rate
FOXO: forkhead box O
GTEx: Genotype-Tissue Expression project
HMOX1 (HO-1): heme oxygenase-1
LD: linkage disequilibrium
MCMC: Markov Chain Monte Carlo
NAD: nicotinamide adenine dinucleotide
PI3K/Akt: phosphatidylinositol 3-kinase/protein kinase B signaling pathway
ROC: receiver operating characteristic curve
SF: synergy factor
SIRT: sirtuin
